# Antimicrobial Effect of a Proteolytic Enzyme From the Fruits of *Solanum granuloso-leprosum* (Dunal) Against *Helicobacter pylori*

**DOI:** 10.3389/fnut.2021.699955

**Published:** 2021-12-16

**Authors:** Ángel Gabriel Salinas Ibáñez, Diego Vallés, Mauricio Adaro, Sonia Barberis, Alba E. Vega

**Affiliations:** ^1^Laboratorio de Microbiología e Inmunología, Facultad de Química, Bioquímica y Farmacia, Universidad Nacional de San Luis, San Luis, Argentina; ^2^Instituto de Física Aplicada (INFAP)-Centro Científico Tecnológico (CCT) San Luis—Consejo Nacional de Investigaciones Científicas y Técnicas (CONICET), San Luis, Argentina; ^3^Laboratorio de Enzimas Hidrolíticas, Facultad de Ciencias, Universidad de la República (UdelaR), Montevideo, Uruguay; ^4^Laboratorio de Control de Calidad y Desarrollo de Bromatología, Universidad Nacional de San Luis, San Luis, Argentina

**Keywords:** granulosain, *Solanum granuloso-leprosum*, safe food additive, antimicrobial proteolytic enzyme, *Helicobacter pylori*, natural adjuvant against *H. pylori*

## Abstract

*Helicobacter pylori* is a gram-negative, helix-shaped, and microaerophilic bacteria that colonizes the human gastric mucosa, causing chronic infections, gastritis, peptic ulcer, lymphomas associated with lymphoid mucosa tissue, and gastric cancer. *H. pylori* is considered a Type 1 human carcinogen by WHO. The prevalence of the infection is estimated in more than half of the world population. Treatment of *H. pylori* infection includes antibiotics and proton pump inhibitors, but the increasing antibiotic resistance promotes the research of novel, more effective, and natural antibacterial compounds. The aim of this work was to study the effect of the partially purified proteolytic extract (RAP) of the fruits from *Solanum granuloso-leprosum* (Dunal), a South American native plant, and a purified fraction named *granulosain* I, against *H. pylori*, to obtain natural food additives for the production of anti-*H. pylori* functional foods. Furthermore, granulosain I and RAP could be used as natural adjuncts to conventional therapies. Granulosain I and RAP antibacterial activity was evaluated as minimum inhibitory concentration (MIC) and minimum bactericidal concentration (MBC) against *H. pylori* NCTC 11638 (reference strain) and twelve *H. pylori* wild strains, using a microdilution plating technique (Clinical and Laboratory Standards Institute). All the strains tested were susceptible to granulosain I with MIC from 156.25 to 312.5 μg/mL and MBC from 312.5 to 625 μg/mL, respectively. Besides, all the strains tested were susceptible to the RAP with MIC from 312.5 to 625 μg/mL and MBC from 625 to 1,250 μg/mL, respectively. The effect of granulosain I and RAP on the transcription of *H. pylori* genes encoding pathogenic factors, *omp*18, *ure*A, and *fla*A, with respect to a housekeeping gene (*16S* rRNA), was evaluated by RT-PCR technique. The band intensity between pathogenic factors and control gene was correlated under treated or untreated conditions, using the ImageJ program. Granulosain I and RAP significantly decreased the expression of pathogenic factors: *omp*18, *ure*A, and *fla*A. The combined inhibitory effect of granulosain I or RAP and an antibiotic such as, amoxicillin (AML, 10 μg), clarithromycin (CLA, 15 μg), levofloxacin (LEV, 5 μg), and metronidazole (MTZ, 5 μg) was evaluated, using the agar diffusion technique. Granulosain I and RAP showed significant synergistic effect on AML, CLA, and LEV, but no significant effect on MTZ was observed. Besides, granulosain I and RAP did not show toxicological effects at the concentrations studied. Finally, granulosain I and RAP could be used as safe natural food additives and as adjuvants for conventional therapies against *H. pylori*.

## Introduction

*Helicobacter pylori* is a gram-negative, curved, and microaerophilic bacillus which are infecting almost 50% of the population. The infection can occur at any time in life but when it occurs in early childhood, a permanent infection is established (1, 2). The bacterium is considered a pathogen of global interest that causes gastritis, peptic ulcer, and lymphomas associated with lymphoid mucosa tissue which are associated with gastric carcinoma (3, 4). The prevalence, age distribution, and the consequences of infection are significantly different in developed and developing countries (5). Since 1994, the International Agency for Research on Cancer (IARC), a subordinate organization of the (WHO), identified *H. pylori* as a group 1 (definite carcinogen) (6).

*H. pylori* remains within the gastric lumen (stomach) for a short time and enters the gastric mucosa, its ecological niche where the pH varies from 4.5 to 6.5. The bacterium has several virulence and pathogenic factors for colonization such as outer membrane proteins, urease, motility, chemotaxis, and its special helix morphology to neutralize the effects of gastric acidic pH (7).

The outer membrane proteins of *H. pylori* such as blood group antigen-binding adhesin (BabA), outer inflammatory protein (OipA), and outer membrane protein (Omp18) are involved in the pathological events of persistent infections, playing critical roles in the metabolism, colonization, pathogenesis, ion transport, adherence, structural and osmotic stability, and antibiotic resistance (7–9).

Besides, *H. pylori* produces large amounts of urease which allows to maintain a constant internal and periplasmic pH, even when the external pH is strongly acidic, preventing the bacteria death and providing a nitrogen source. For this reason, the synthesis of urease has been a well-studied therapeutic target in the literature (10).

The urease activity and motility of *H. pylori* are relevant pathogenic factors for the initial bacterial colonization. If the motility decreases, the ability to colonize or survive in the host also decreases (7, 8).

The bacterial flagellum is a complex motility organ composed of several types of protein subunits, among them the flagellin protein encoded by *fla*A (11, 12). Several authors reported that the helical shape and motility of *H. pylori* strains are correlated with the degree of infectivity and the colonization ability (7, 13, 14).

Standard first-line treatment for *H. pylori* infection includes the use of antibiotics clarithromycin (CLA), metronidazole (MTZ), or amoxicillin (AML) and proton pump inhibitors, for 14 days to increase the effectiveness of eradication (15). However, the antibiotic resistance of *H. pylori* has reached alarming levels worldwide and is the leading cause of treatment failure (16–18). The WHO promoted an urgent search for new efficient compounds for the priority treatment of 16 multiresistant microorganisms, including *H. pylori* (19).

New effective and safe natural antibacterial compounds that can act on the *H. pylori* targets or modulate the host's immune system are actively sought. The use of plant extracts and their derivatives constitutes an emerging therapeutic approach (20, 21).

Polyphenols from wine, apple peel, or olive oil allowed to decrease the secretion of some virulence factors such as urease and adhesion molecules (SabA, VacA) and caused disruption of the outer membrane of some bacteria (22). Bacteria exposed to adverse environments can upregulate the genes transcription for survival and virulence (23).

Proteolytic enzymes are a diverse group of enzymes that differ in structure, substrate specificity, and reaction mechanism (24, 25). Several authors reported that subtilisin was effective against Pseudomonas sp, Bacillus sp, and *Listeria monocytogenes* (26, 27); lysostaphin endopeptidase from *Staphylococcus simulans* which lyses cell walls by cleaving the pentaglycine linkage between the peptidoglycan chains (28), whereas pronase from *Streptomyces griseus* did not show an additive effect on the eradication of *H. pylori* infection after the patients received the standard triple therapy, which consists of a proton pump inhibitor with AML and CLA two times a day for 7 days (29).

Recent studies have shown that the partially purified proteolityc extract (≥ 50 μg of protein/mL) of the fruits from *Solanum granuloso-leprosum* (Dunal), a South American native plant, was able to elicit a significant decrease (*p* ≤ 0.05) in the growth of *Escherichia coli* ATCC 25922, but no effect was observed on the growth *Staphylococcus aureus* ATCC 25923 (30). However, there is little information on the isolation of proteases from native plants (31–36), and their antimicrobial activity against *H. pylori* has not been studied.

Besides, the combined synergistic effects of herbal drugs or phytochemicals with synthetic antibiotics are a new strategy to prevent the development of resistance (37). Some studies have shown that plant-derived compounds can act in synergy with various antibiotics against resistant pathogens, including *H. pylori*, improving the eradication rate and increasing the sensitivity of multidrug-resistant bacteria (38, 39).

The aim of this work was to study the effect of the partially purified proteolytic extract (RAP) of the fruits from *Solanum granuloso-leprosum* (Dunal), a South American native plant, and a purified fraction named *granulosain* I, against *H. pylori*, to obtain natural food additives or ingredients for the production of anti-*H. pylori* functional foods. Furthermore, granulosain I and RAP could also be used as natural adjuncts to conventional therapies.

## Materials and Methods

### Plant Material

Samples of plant material were deposited in the Uruguayan Botanical Garden Museum “Prof. Atilio Lombardo,” which is part of the Municipality of Montevideo, Uruguay. The samples of this species were cataloged and inventoried by the Museum as N° MVJB-9276 for *Solanum granuloso-leprosum* (Dunal).

### Preparation of the Partially Purified Granulosain Extract and Purification of Granulosain I

Fresh mature fruits were washed, dried, and grinded in a mortar. Homogenates were filtered through a piece of gauze folded in two to remove plant debris and then centrifuged for 15 min at 6,654 × g (Refrigerated Centrifuge, Model 5430R Eppendorf, Medical Equipment Specialists Inc., Ma, USA). The supernatant was treated with four volumes of cold (−20°C) acetone with gentle agitation and left to settle for 20 min before centrifugation at 6,654 × g for 30 min. The final acetone precipitate was dissolved with one volume of 50 mM buffer Tris–HCl pH 7.5 (4.9 ± 0.01 mg of protein/mL) and lyophilized (Lyophilizer EQL-01497, BK-FD10P Model, Dauerhaft Brand, Biobase Biotech Co. Ltd., Jinan, China), and it was named *partially purified granulosain extract*. The protein content was determined by Bradford protein assay (40). The purification of granulosain I was carried out by ion-exchange chromatography on a HiTrap SP HP column (Meck KGaA, Darmstadt, Alemania) according to the procedure described by Vallés et al. (41).

Before bioactivity assays were performed, granulosain I was redissolved in distilled water to get a stock solution (0.26 ± 0.01 mg of protein/mL), which was sterilized by filtration through MF-Millipore nitrocellulose membranes, pore size 0.22 μm, diameter 47 mm. Several serial dilutions of the purified enzyme (granulosain I), from 3.875 μg of protein/mL to 497 μg of protein/mL, were prepared. Similarly, several dilutions of the partially purified granulosain extract (RAP), from 19.55 μg of protein/mL to 2,500 μg of protein/mL, were prepared from the stock solution and they were used in all tests.

### Electrophoresis

Native PAGE electrophoresis was performed in gels composed by a stacking gel (*T* = 4.5%) and a running gel (*T* = 12.5%). After loading samples, the electrophoresis run was carried out at a constant current of 90 V, and then, it was increased to 100 V and kept constant for 90 min. The gels were stained with Coomassie brilliant blue-R-250 dissolved in a solution of 50% (v/v) ethanol and 10 % (v/v) acetic acid, and they were decolorized with the same solution to detect protein bands.

### Zymogram

The unstained gel of the native PAGE was previously incubated in 0.1 M Tris–HCl buffer pH 7.5 with 15 mM cysteine during 15 min. Then, it was placed on an agarose gel (imbibed in 1% casein solution), avoiding the formation of bubbles. The assembly was placed in a humid chamber and heated to 50°C for 15 min to detect proteolytic activity bands. After incubation, the agarose gel was fixed with a solution of 50% (v/v) ethanol and 10% (v/v) acetic acid for 30 min, dehydrated, and stained with Coomassie brilliant blue R-250.

### Protein Concentration and Specific Proteolytic Activity

Protein concentration of the partially purified extract and granulosain I was determined by Bradford protein assay, with bovine albumin as protein standard (40). The specific proteolytic activity of those enzyme extracts was measured during purification steps, using azocasein as substrate. The reaction mixture consisting of 340 μL of 1% w/v azocasein solution (in 0.1 M Tris–HCl buffer pH 7.5 containing 15 mM Cys), 340 μL of diluted enzyme extracts (RAP or granulosain I) which were previously activated with 15 mM Cys (final concentration), and 340 μL of 0.1 M Tris–HCl buffer pH 7.5 containing 15 mM Cys), was incubated for 10 min at 37°C. The enzymatic activity was stopped for the addition of 340 μL of 10% w/v TCA, the mixture was centrifuged for 20 min at 15,400 × g, and the absorbance of the supernatant was measured at λ: 337 nm (Cintra 2020 UV–vis spectrometer, GBS Scientific Equipment Pty Ltd., Braeside, Victoria 3195, Australia). The unit of enzymatic activity (Azocaseinolytic Unit, Uazo) is defined as the amount of enzyme that produces an increase of one absorbance unit measured at λ: 337 nm after 1 min, under the test conditions. As a negative control, the activity assay was performed with buffer solution instead of enzyme extracts.

### Physicochemical Properties of the Partially Purified Extract and Granulosain I

#### Stability Upon Storage

The residual proteolytic activity of the enzyme extracts (RAP or granulosain I) during its storage at −20°C was monitored each 24 h for 24 months, using N-α-benzoyl-DL-arginine-p-nitroanilide (BAPNA) (Sigma-Aldrich, USA) as substrate. 0.5 mL of enzyme extracts (RAP or granulosain I) and 0.5 mL of 10 mM BAPNA with 20 mM L-cysteine in 0.1M Tris–HCl buffer pH 8 were mixed and incubated at 37°C under 200 rpm of agitation, during 5 min. The change in absorbance was measured at λ: 410 nm during 5 min, within the linearity range. One international unit (IU) of the proteolytic activity of the enzyme extracts was established as the amount of enzyme that cleaves 1 μmol of BAPNA per min under previously mentioned conditions. Controls under similar conditions but without substrate were also carried out.

#### Solubility

Enzyme solubility was defined as the amount of soluble nitrogen that results after applying a specific procedure. The protein content in the sample and in the supernatant was quantified by the Kjeldahl method (42). This property provides information on the ability of the enzyme extracts (RAP and granulosain I) to form colloidal solutions and depends on pH, ionic strength, and temperature (43). Solubility is expressed as protein solubility index (PSI) (44), as follows (equation 1):


(1) $$\begin{array}{r} \text{PSI}=\;\frac{\text{Protein content in the supernatant (mg/mL)}\times \text{Volume of supernatant (mL)}}{\text{Sample weight (mg)}\times \text{Protein content in the sample (mg/100 mg of sample)}}\times \text{100} \end{array}$$

#### Emulsifying Properties

The enzyme content needed to coat an interfacial area is related to the ability to create and stabilize an emulsion. The emulsifying activity index (EAI, m^2^/g) and the emulsion stability index (ESI, min) of enzyme extracts (RAP or granulosain I) were determined by the turbidimetric method of Pearce and Kinsella (45) modified by Tang et al. (46). 2 mL of the enzymatic solution dissolved in 18 mL of 50 mM Tris–HCl buffer pH 7.5 (10 mg of protein/mL) was added to 6 mL of corn oil (θ: 0.23) and stirred for 3 min at maximum speed, in a homogenizer (HH-S-1000, Hermann, Argentina). 50 μl of the emulsion was poured onto 2.5 mL of 0.1% SDS (DF: 51), mixed in a vortex, and the absorbance at λ: 500 nm was immediately measured (A_0_) and after 20 min (A_20_), using a Cintra 2020 UV–vis spectrometer (GBS Scientific Equipment Pty Ltd., Braeside, Victoria 3195, Australia). The EAI and ESI were calculated by means of equations (2) and (3):


(2) $$\begin{array}{r} \text{EAI}=\frac{2\text{ x }2,303\times \text{Ao}\times \text{DF}}{\text{cx ϕ}\times \left(1-\text{θ}\right)} \end{array}$$

(3) $$\begin{array}{r} \text{ESI}=\frac{{\text{A}}_{0}}{\left({\text{A}}_{0}-{\text{A}}_{20}\right)}\times 20 \end{array}$$

Where:

c: is the protein concentration (mg/mL).

ϕ: is the optical path (0.01 m).

θ: is the fraction of oil used to form the emulsion.

DF: is the dilution factor.

#### Viscosity

The viscosity of enzyme extracts (RAP or granulosain I, 10 mg of protein/mL) was determined with a programmable rheometer (Model DV-III, AMETEK Brookfield, MA, USA). Routines of 9 measurements each were carried out at different revolutions (from 100 to 140 rpm) and at 24°C and the mean viscosity was calculated (47).

#### Hydration Properties

The ability of proteins to interact with water influences food formulation, processing, and storage. The hydration properties of the enzyme extracts (RAP or granulosain I) were measured as held water (HW, %) and as water retention capacity (WHC, g of water/g of dry residue) (48), based on the amount of water that a protein can retain under centrifugation at 3,000 × g for 30 min at 20°C (Refrigerated Centrifuge, Model 5430R Eppendorf, Medical Equipment Specialists Inc., Ma, USA), according to equations (4) and (5).


(4) $$\begin{array}{r} \text{HW}=\frac{\text{weight of }{\text{H}}_{2}\text{O in pellet}}{\text{weight of }{\text{H}}_{2}\text{O in pellet}+\text{weight of }{\text{H}}_{2}\text{O in supernatant}} \end{array}$$

(5) $$\begin{array}{r} \text{WHC}=\frac{\text{weight of }{\text{H}}_{2}\text{O in pellet}}{\text{weight of dry pellet}}\times 100 \end{array}$$

### Bacterial Strains

Thirteen *H. pylori* strains were assayed in this study. *Helicobacter pylori* NCTC 11638 (The National Collection of Type Cultures, Culture Collections UK Health Security Agency, Porton Down Salisbury SP4 0JG UK) (sensitive to AML, MTZ, LEV and CLA) was used as reference strain. It was a kind gift from Dr. Teresa Alarcon Cavero, Microbiology Service of Hospital Universitario de la Princesa (Madrid, Spain). Besides, twelve wild strains isolated from gastric antral biopsy specimens of patients in the Central Microbiology Laboratory of the San Luis Ministry of Health, Government of San Luis, Province of San Luis, Argentina were also used. They were identified as follows:

- *Helicobacter pylori* HP 109 (sensitive to AML, CLA, LEV, and with natural or intrinsic resistant to MTZ). It was obtained from a gastric antral biopsy specimen of patient with chronic gastritis.
- *Helicobacter pylori* HP 137 (sensitive to AML, MTZ, LEV, and with natural or intrinsic resistant to CLA). It was obtained from a gastric antral biopsy specimen of patient with chronic gastritis.
- *Helicobacter pylori* HP 145 (sensitive to AML, CLA, LEV, and with natural or intrinsic resistant to MTZ). It was obtained from a gastric antral biopsy specimen of patient with duodenal ulcer.
- *Helicobacter pylori* HP 148 (sensitive to AML, MTZ, LEV, and with natural or intrinsic resistant to CLA). It was obtained from a gastric antral biopsy specimen of patient with gastric ulcer.
- *Helicobacter pylori* HP 152 (sensitive to AML and LEV and with natural or intrinsic resistant to CLA and MTZ). It was obtained from a gastric antral biopsy specimen of patient with gastric ulcer.
- *Helicobacter pylori* HP 155 (sensitive to AML, MTZ, LEV, and CLA). It was obtained from a gastric antral biopsy specimen of patient with chronic gastritis.
- *Helicobacter pylori* HP 166 (sensitive to AML, MTZ, LEV, and CLA). It was obtained from a gastric antral biopsy specimen of patient with chronic gastritis.
- *Helicobacter pylori* HP 179 (sensitive to AML, MTZ, LEV, and with natural or intrinsic resistant to CLA). It was obtained from a gastric antral biopsy specimen of patient with chronic gastritis.
- *Helicobacter pylori* HP 294 (sensitive to AML and LEV and with natural or intrinsic resistant to CLA and MTZ). It was obtained from a gastric antral biopsy specimen of patient with duodenal ulcer.
- *Helicobacter pylori* HP 659 (sensitive to AML, MTZ, LEV, and CLA). It was obtained from a gastric antral biopsy specimen of patient with chronic gastritis.
- *Helicobacter pylori* HP 661 (sensitive to AML, CLA, MTZ, and with natural or intrinsic resistant to LEV). It was obtained from a gastric antral biopsy specimen of patient with gastric ulcer.
- *Helicobacter pylori* HP 662 (sensitive to AML, CLA, LEV, and with natural or intrinsic resistant to MTZ). It was obtained from a gastric antral biopsy specimen of patient with chronic gastritis.

The strains were grown in Mueller-Hinton agar (MHA, Britania, Buenos Aires, Argentina) supplemented with 7% horse blood (MHA-B) and incubated in microaerophilia (O_2_ 5%, CO_2_ 10%, and N_2_ 85%) at 37°C, for 2 or 3 days. The storage of strains was carried out at −20 and −80°C in trypticase soy broth (TSB, Britania) supplemented with 20% glycerol (Biopack, Buenos Aires, Argentina).

The *H. pylori* strains were positively identified by microscopy, and urease, catalase, and oxidase tests. In addition, the sensitivity of the strains was evaluated by the MIC breakpoint values, being ≤ 25 mm for AML (10 μg/mL), ≤ 28 mm for CLA (15 μg/mL), ≤ 18 mm for MTZ (5 μg/mL), and ≤ 18 mm for LEV (5 μg/mL) (49).

### Planktonic Cultures of *H. pylori*

Planktonic cultures were obtained by inoculating 1 mL of a *H. pylori* suspension adjusted to 0.5 turbidity McFarland standard (c.a. 1 ×10^8^ colony forming units (CFUs / mL) in Petri dishes containing 14 mL of Mueller-Hinton broth (MHB, Britania, Buenos Aires, Argentina) added with 5% fetal calf serum (Gibco^TM^). Petri dishes were incubated under the previously mentioned conditions.

### Antibacterial Activity of the Partially Purified Extract and Granulosain I

The antibacterial activity of the enzyme extracts (RAP and granulosain I) against thirteen *H. pylori* strains was assessed as minimum inhibitory concentration (MIC) and minimum bactericidal concentration (MBC) using the broth microdilution method according to CLSI guidelines (50).

The broth microdilution method was carried out in polystyrene 96-well microtiter plates. First, 2-fold serial dilutions of RAP (from 19.55 to 2,500 μg of protein/mL, which is from 0.05 to 6.5 Uazo/mL) and granulosain I (from 3.875 to 497 μg of protein/mL, which is from 0.05 to 6.5 Uazo/mL) were performed in MHB. Then, a 100 μl aliquot of each enzyme extract dilution was added to 100 μl MHB broth, and 15 μl of each bacterial suspension adjusted to a scale of 0.5 on the McFarland standard (1 ×10^8^ CFU./mL) was dispensed in each well of the microtiter plate. Next, a 5 mg/mL of 2,3,5-triphenyl tetrazolium chloride (TTC, Merck KGaA, Darmstadt, Germany) solution was added as a viability indicator. TTC was previously dissolved in sterile distilled water at room temperature and the solution was filtered through a 0.22-mm filter and stored at −70°C until needed. Plates were incubated under the microaerobic condition at 37°C. After 72-h incubation, the plates were inspected for visual color change; the results were recorded and interpreted. Living cells turn red and dead cells remain colorless.

The MIC was determined as the lowest concentration of the enzyme extract (RAP or granulosain I) required to inhibit the visible growth of the microorganism.

A growth positive control was made with 100 μl of MHB broth and 15 μl of the bacterial suspension, and it was incubated under the same growth condition.

A negative control with 100 μl of MHB broth and 15 μl of 0.9% normal saline solution was also incubated under the same growth conditions, and it served as a control for contamination, indicated by an absence of color change.

Minimum bactericidal concentration (MBC) was defined as the least concentration of the enzyme extract (RAP or granulosain I) that prevented the growth of bacteria on antibiotic-free culture media. The MBC was determined from broth dilution MIC tests by subculturing to MHA-B agar plates that did not contain the enzyme extracts. Thus, 5 μl of broth was taken from each well where no visible growth was observed and streaked on MHA-B. These plates were incubated at the same conditions that those established for MIC.

### Effects of Subinhibitory Concentrations (Sub-MICs) of the Partially Purified Extract and Granulosain I on the Transcription (Expression) of *H. pylori* Genes Encoding Pathogenic Factors

The sub-MICs (½ MIC) of the enzyme extracts (RAP and granulosain I) were used in the genes' expression and synergism assays.

Total RNA was isolated from planktonic cultures of thirteen strains, before and after treatment with the enzyme extracts, using the TRIZOL reagent according to the manufacturer's instructions (Invitrogen, Buenos Aires, Argentina). The isolated RNA was stored at −20°C. The cDNA was obtained from a reaction mixture including random hexamers and 200 U Moloney Murine Leukemia Virus Reverse Transcriptase (M-MLV RT, Invitrogen, Buenos Aires, Argentina) and stored at −20°C.

Transcription levels of pathogenic factors such as *omp*18, *ure*A, and *fla*A were determined by RT-PCR. The 16S *rRNA* amplicon was used as housekeeping.

The PCR amplification was performed in a programmable thermal cycler (BioRad, California, USA), using the primer pairs shown in Table 1 and the protocols described in Table 2. The identification of RT-PCR products was performed with 1.8% agarose gel electrophoresis. The gels were stained with GelRed Nucleic Acid Gel Stain (Biotium Inc. Hayward, CA, USA), visualized under UV light, and photographed. A 100-bp DNA ladder (PBL, Quilmes, Buenos Aires, Argentina) was included as a molecular mass reference. The semiquantification of the DNA amplicons was performed using software of image processing and analysis in Java (ImageJ, Maryland, USA).

**Table 1:** Primers used for RT-PCR targeted to *Helicobacter pylori* genes.

**Primer**	**Primer sequence (5^**′**^-3^**′**^)**	**Size amplicon (bp)**
16S *rRNA*-F	GGAGGATGAAGGTTTTAGGATTG	390
16S *rRNA*-R	TCGTTTAGGGCGTGGACT	
*omp*18-F	TGCTTTTGGAAGGCAATACC	165
*omp*18-R	CATTTGGGTTTGGTTTCACC	
*ure*A-F	GCCAATGGTAAATTAGTT	411
*ure*A-R	CTCCTTAATTGTTTTTAC	
*fla*A -F	GTGGCGCAAAAAGTGGCTAA	237
*fla*A-R	GTAATCGGCCGGTTTCAAGC	

**Table 2:** Protocols of PCR amplification for *Helicobacter pylori* genes.

**Steps**	**Temperature (**°**C)**	**Time (min)**
**16S** ***rRNA*** **and** ***omp*****18 genes**		
Initial desnaturalization	94	3
30 cycles	94	1
	58	1
	72	1
Final extensión	72	10
***ureA*** **and** ***fla*****A genes**		
Initial desnaturalization	95	5
	94	1
35 cycles	45	1
	72	1
Final extensión	72	7

### Synergistic Effect of Combining Antibiotics and the Partially Purified Extract and Granulosain I

The synergism assays between the enzyme extracts (RAP or granulosain I) and each antibiotic were evaluated by the disk diffusion method on MHA-B media (51). Synergistic effect was determined as the increase in the diameter of the halos (mm).

Four antibiotics such as amoxicillin (AML, 10 μg), clarithromycin (CLA, 15 μg), metronidazole (MTZ, 5 μg) (Oxoid^TM^, Argentina), and levofloxacin (LEV, 5 μg) (Britania Laboratories, Argentina) were evaluated in combination with the enzyme extracts (RAP or granulosain I).

Thirteen sets of antibiograms were performed in triplicate; that is, one set for each *H. pylori* strain. Each set consists of plating a *H. pylori* strain in MHA-B containing each antibiotic alone, and similar cultures combined with sub-MIC of the enzyme extracts (RAP or granulosain I). The diameter (mm) of each inhibition zone was recorded after incubation for three days under the previously mentioned conditions.

### Cytotoxicity Assays

#### Laboratory Animals

BALB/c wild-type (WT) mice of 20 g of body weight (40 days old) were provided by the Animal Facility of the National University of San Luis, San Luis, Argentina; they were maintained with food and water *ad-libitum*. The animals were handled and cared according to the regulations of the Institutional Committee for the Care and Use of Animals of the National University of San Luis (CICUA-UNSL), San Luis, Argentina, and the Guides for the Care and Use of Laboratory Animals (52).

Three groups of animals received intragastric administration of phosphate-buffered saline (PBS) or enzyme extracts (RAP or granulosain I) to evaluate their potential hepatotoxicity and nephrotoxicity. The first group was treated with 1 × PBS. The second group was treated with three doses of 250 μl of RAP (625 μg/mL) at intervals of 48 h. The third group was treated with three doses of 250 μl of granulosain I (312.5 μg/mL) at intervals of 48 h, under the same conditions to the other animal groups. After three days, the animals were killed and the serum was obtained for further studies.

#### Activity of Aspartate Aminotransferase, Alanine Aminotransferase, and Creatinine in Serum

The AST, ALT, and creatinine activity were determined in treated and non-treated mice serum with transaminases 200 and creatinina commercial kit (Wiener Lab, Rosario, Santa Fé, Argentina), according to the manufacturer's instructions. The aminotransferase and creatinine activities were expressed as IU/L and mg/l, respectively (53).

#### Hepatotoxicity Assay Using Transaminases (AST and ALT)

The AST and ALT assays are based on the reaction of pyruvate with 2,4-dinitrophenylhydrazine which produces a colored compound in an alkaline medium that is measured at λ: 505 nm. In a hemolysis tube, 250 μl of the substrate was placed and incubated in a 37°C water bath for a few min. Then, 50 μl of serum was added, mixed, and incubated for 30 min, and 500 μl of 2,4-dinitrophenylhydrazine was added. The reaction mixture was homogenized and left 10 min at 37°C. Finally, 2.5 mL of enzyme diluent was added and mixed and after 2 min, the absorbance was read at λ: 505 nm (Cintra 2020 UV–vis spectrometer (GBS Scientific Equipment Pty Ltd., Braeside, Victoria 3195, Australia).

#### Nephrotoxicity Assay by Creatinine Activity

The assay is based on the reaction of creatinine with alkaline picrate in a buffered medium to obtain a chromogenic compound which is measured at λ: 510 nm. In a hemolysis tube, 100 μl of serum and 500 μl of reagent 1 (41.4 mmol/L picric acid) were added, mixed, and allowed to stand for 10 min. The tube was centrifuged at 3,000 rpm for 5 min. Then, 375 μl of supernatant was taken out and 63 μl of reagent 2 (glycine buffer/ 1.0 M NaOH) was added. The tube was mixed by inversion and incubated for 20 min at room temperature. The reaction mixture was measured in a spectrophotometer at λ: 510 nm (Cintra 2020 UV–vis spectrometer (GBS Scientific Equipment Pty Ltd., Braeside, Victoria 3195, Australia).

### Statistical Analysis

Specific enzyme activity, protein content, and physicochemical properties of the enzyme extracts were calculated from three separate assays which were performed in duplicate, and the results were informed as mean ± standard deviation (SD). The linear region of the reaction progress of enzyme activity was also determined. The determination of MIC and MBC was done in triplicate. The genes' expression, synergism, and cytotoxicity assays were performed as three separate assays by duplicate, and the results were expressed as mean ± SD. Statistical analysis was made with InfoStat/L Statistical Software for Windows (Universidad Nacional de Córdoba, Córdoba, Argentina). A probability of *p* < 0.05 was considered significant according to the Student's *t*-test.

## Results and Discussion

### Proteolytic Extract of Fruits From Solanum Granuloso-Leprosum and the Main Purified Fraction (Granulosain I)

The crude extract of *S. granuloso-leprosum* fruits contains high concentrations of sugars, pigments, phenols, and vitamin C (54). The partially purified proteolytic extract of fruits from *Solanum granuloso-leprosum* was obtained by means of protein acetone precipitation. The main purified fraction was named granulosain I (23.878 kDa) (41).

**Figure 1:**
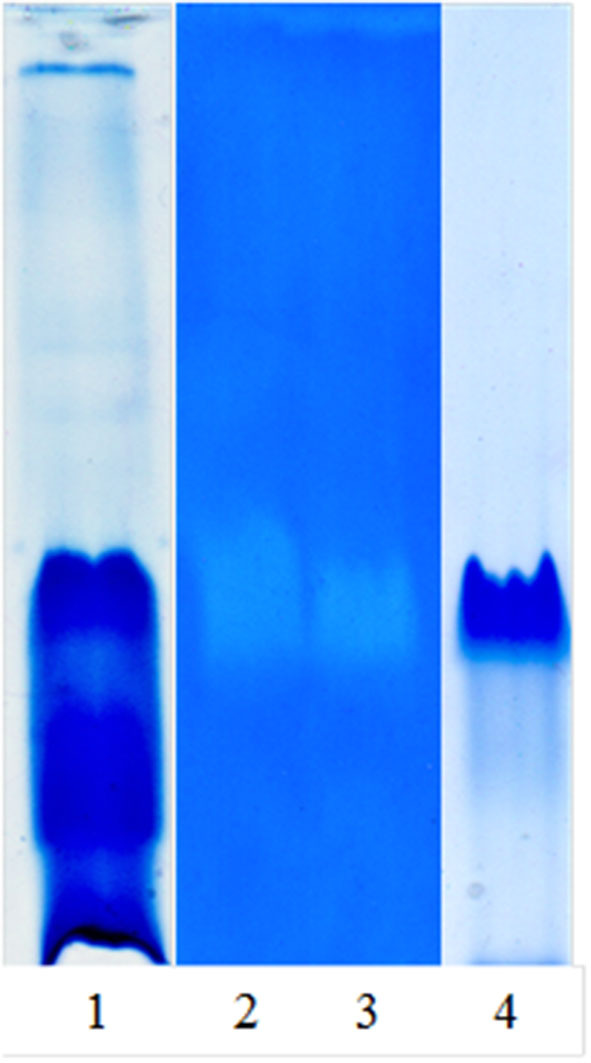
Native PAGE and zymogram of partially purified proteolytic extract (RAP) of *Solanum granuloso-leprosum* and granulosain I. Lane 1, RAP, stained gel with Coomassie brilliant blue-R-250; Lane 2, zymogram of RAP; Lane 3, zymogram of granulosain I; Lane 4, granulosain I, stained gel with Coomassie brilliant blue-R-250.

Figure 1 shows the native PAGE and zymogram of the partially purified extract and the main purified fraction (granulosain I). As shown in Lane 4, a high purification degree was obtained after using ion-exchange chromatography on a Hi-trap SP HP column.

The partially purified proteolytic extract and granulosain I showed high specific proteolytic activity in 0.1 M Tris–HCl buffer pH 7.5 containing 15 mM Cys, being 2.6 ± 0.01 U_azo_/mg of protein and 13.1 ± 0.00 U_azo_/mg of protein, respectively. The protein content of the partially purified extract and of granulosain I was 4.9 ± 0.01 mg/mL and 0.26 ± 0.01 mg/mL, respectively.

### Physicochemical Properties of the Partially Purified Extract and Granulosain I

This work focuses on the production of anti-*H. pylori* functional foods, which according to our knowledge are not yet available on the market. Although food additives such as RAP or granulosain I will be added in low concentration to different foods, they must be stable and have certain physicochemical characteristics that do not alter the properties of the final product.

The partially purified proteolytic extract (RAP) and granulosain I showed very high storage stability at −20°C, since the residual proteolytic activity did not change for 24 months. In addition, the RAP extract showed low solubility (PSI: 2.7%), low viscosity (μ: 1.33 cP), low water retention (HW: 3.45% or WHC: 6.79 g of H_2_O/g of dry residue), and good emulsifying properties (EAI: 1,108 m^2^/g, ESI: 34.7 min), if its physicochemical properties are compared to common liquids for food use (55–57). Besides, the physicochemical properties of granulosain I showed similar physicochemical properties than the RAP extract, and no significant differences (*p* < 0.05) were found between them.

### Antibacterial Activity of the Partially Purified Extract and Granulosain I

The antibacterial activity of the partially purified proteolytic extract (RAP) and granulosain I was evaluated as MIC and MBC against *H. pylori* NCTC 11638 (reference strain) and twelve wild *H. pylori* strains isolated from gastric antral biopsy specimens of patients in the Central Microbiology Laboratory of the San Luis Ministry of Health, Government of San Luis, Province of San Luis, Argentina. The susceptibility of each strain and the pathologies associated with them were described in section Bacterial Strains.

As shown in Table 3, all *H. pylori* strains tested were susceptible to granulosain I with MIC values from 156.25 to 312.5 μg/mL and MBC values from 312.5 to 625 μg/mL, respectively. Besides, all the strains tested were susceptible to RAP with MIC values from 312.5 to 625 μg/mL and MBC values from 625 μg/mL to 1,250 μg/mL, respectively.

**Table 3:** MIC and MBC of the partially purified proteolytic extract (RAP) of the fruits from *Solanum granuloso-leprosum* and its main purified fraction (granulosain I) against *H. pylori* strains by means of the broth microdilution method.

***H pylori* strains**	**Pathology**	**RAP**		**Granulosain I**	
		**MIC (μg/mL)**	**MBC (μg/mL)**	**MIC (μg/mL)**	**MBC (μg/mL)**
**Sensitive to AML, MTZ, LEV, and CLA**					
NCTC 11638	Reference	625.0	625	312.5	312.5
HP155	Chronic gastritis	312.5	625	156.25	312.5
HP166	Chronic gastritis	312.5	625	156.25	312.5
HP179	Chronic gastritis	312.5	625	156.25	312.5
HP659	Chronic gastritis	625.0	625	312.50	312.5
**Single-drug resistant**					
HP109	Chronic gastritis	312.5	625	156.25	312.5
HP137	Chronic gastritis	312.5	625	156.25	312.5
HP145	Duodenal ulcer	625.0	1,250	312.50	625.0
HP148	Gastric ulcer	312.5	625	156.25	312.5
HP661	Gastric ulcer	625.0	625	312.50	312.5
HP662	Chronic gastritis	625.0	625	312.50	312.5
**Multidrug-resistant**					
HP152	Gastric ulcer	625.0	1,250	312.5	625.0 0
HP294	Duodenal ulcer	625.0	1,250	312.5	312.5

*The values were expressed as mean of the experiments in triplicate (n = 3). No visual color difference was observed between triplicate tests performed under the same conditions*.

The MBC of granulosain I is 312.5 μg/mL for all *H. pylori* strains studied, regardless of their sensitivity to antibiotics (CLA, LEV, MTZ, and AML), except for *H. pylori* HP 145 resistant to MTZ and for *H. pylori* HP 152 resistant to MTZ and CLA. Granulosain I against those stains, *H. pylori* HP 145 and HP 152, doubled the value of MBC (625 μg/mL). However, similar behavior was not observed against *H. pylori* HP 109 and HP 662, both resistant to MTZ, and against *H. pylori* HP 294 resistant to MTZ and CLA.

Although the enzyme extracts (RAP and granulosain I) were prepared at the same enzymatic activity (Uazo/mL), higher MIC and MBC values were exhibited for the RAP. It is likely that the higher content of proteins without proteolytic activity and other components of the RAP have affected the interaction between the active protease and the microbial cell surface, decreasing the antibacterial activity of the RAP with respect to the purified fraction. These results allow to justify that granulosain I, the main protease of the proteolytic extract of the fruits of *S. granuloso-leprosum*, is responsible for the antibacterial activity against the *H. pylori* strains.

### Effects of Subinhibitory Concentrations (Sub-MICs) of the Partially Purified Extract and Granulosain I on the Transcription (Expression) of *H. pylori* Genes Encoding Pathogenic Factors

The effect of subinhibitory concentrations (sub-MICs) of the partially purified proteolytic extract (RAP) (156.25 μg/mL) and granulosain I (78.12 μg/mL) on the expression of the *H. pylori* genes encoding pathogenic factors, such as *omp*18, *ure*A and *fla*A genes, was determined by RT-PCR.

**Figure 2:**
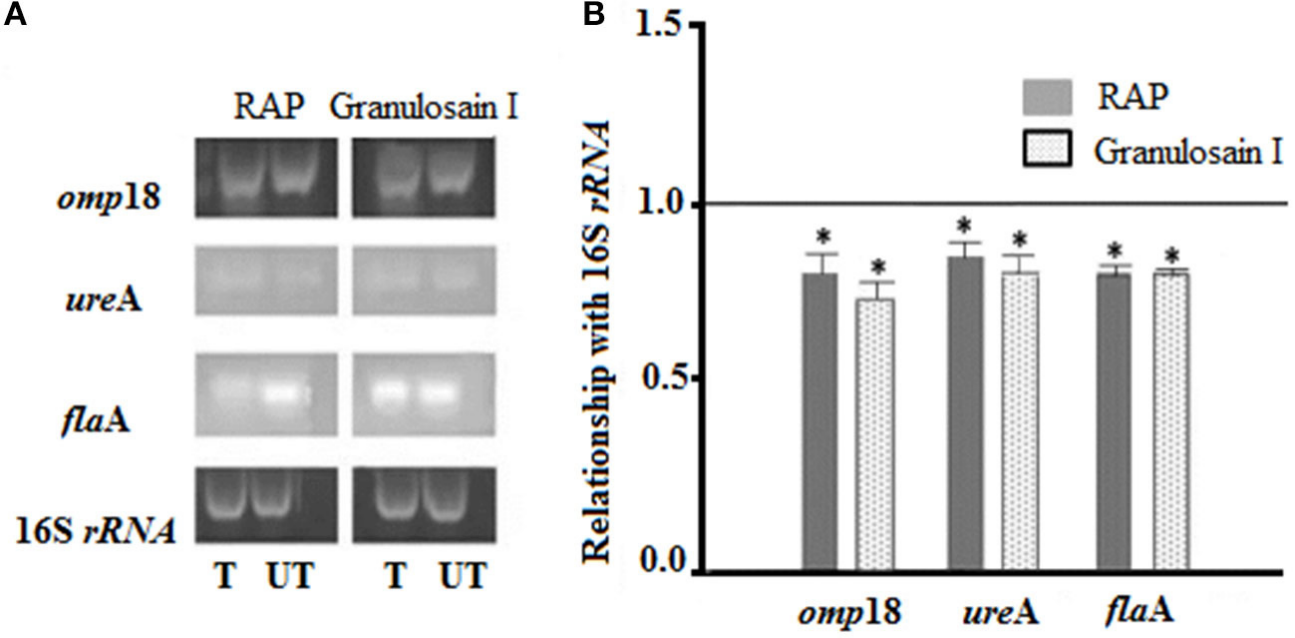
**(A)** Electrophoretic gels: *Helicobacter pylori* amplicons resulting from RT-PCR in 1.8% agarose gels stained with Gel Red®. (T) Treated cultures and (UT) untreated cultures of *H. pylori* with the partially purified proteolytic extract (RAP) of the fruits of *Solanum granuloso-leprosum* and granulosain I. **(B)** Relative quantification of the expression of the *H. pylori* pathogenic factors, before and after treating *H. pylori* cultures with RAP and granulosain I. *Represents significant differences (*p* ≤ 0.05) with respect to 16S *rRNA*, according to the Student's *t*-test.

Amplicons of planktonic *H. pylori* cultures, which were grown in the presence (T, treated cultures) and in the absence (UT, untreated cultures) of the enzyme extracts (RAP and granulosain I), are shown in Figure 2.

The expression levels of the *omp*18, *ure*A, and *fla*A pathogenic factors obtained from treated and untreated cultures were normalized, using the expression level of 16S rRNA gene (value 1). A comparison of the normalized gene expression between treated and untreated cultures was made and those values were graphed. The mRNA expression levels in *omp*18, *ure*A, and *fla*A significantly decreased in treated cultures (*p* < 0.05).

According to the literature, resveratrol was able to downregulate the expression of the *omp*18 gene (outer membrane protein gene), but the expressions of the *ureA* (urease) and *fla*A (flagellin) genes were upregulated (58). Celecoxib inhibited *H. pylori* motility by decreasing mRNA expression of the *fla*A gene, but the *ure*A gene was upregulated (59). The aqueous extracts of *Litrahea molleoides* and *Aristolochia argentina*, two native plants of the Province of San Luis, Argentina, were able to downregulate the expression of the *ure*A gene from *H. pylori* cultures (20). Several authors have reported that honey, polyphenols, flavonoids, thiourea, dihydroxyacetone, and sulforaphane were able to inhibit the urease activity (60–62).

According to our results, the enzyme extracts (RAP and granulosain I) of *S. granuloso-leprosum* fruits could reduce *H. pylori* colonization and attenuate the pathogenesis in gastric mucosa. In fact, the downregulation effect of *omp*18 by the RAP and granulosain I would influence on the bacterial adherence, nutrient uptake, and induction of inflammation in the gastric mucosa. Furthermore, the RAP and granulosain I were able to downregulate *H. pylori* motility and the expression of its urease, which allowed to decrease nitrogen supply for bacterial growth and hindered bacterial adaptation to the digestive tract.

### Evaluation of Synergistic Effect of Combining Antibiotics and the Partially Purified Extract and Granulosain

Thirteen sets of antibiograms were performed in triplicate, which is one set for each *H. pylori* strain. Each set consists of plating a *H. pylori* strain in MHA-B containing each antibiotic alone and incubating for three days under the conditions mentioned above (Control); similar cultures combined with sub-MIC of the enzyme extracts (RAP or granulosain I) to evaluate the synergistic effects. The diameter of the halo (mm) of each zone of inhibition was recorded and comparatively analyzed.

**Figure 3:**
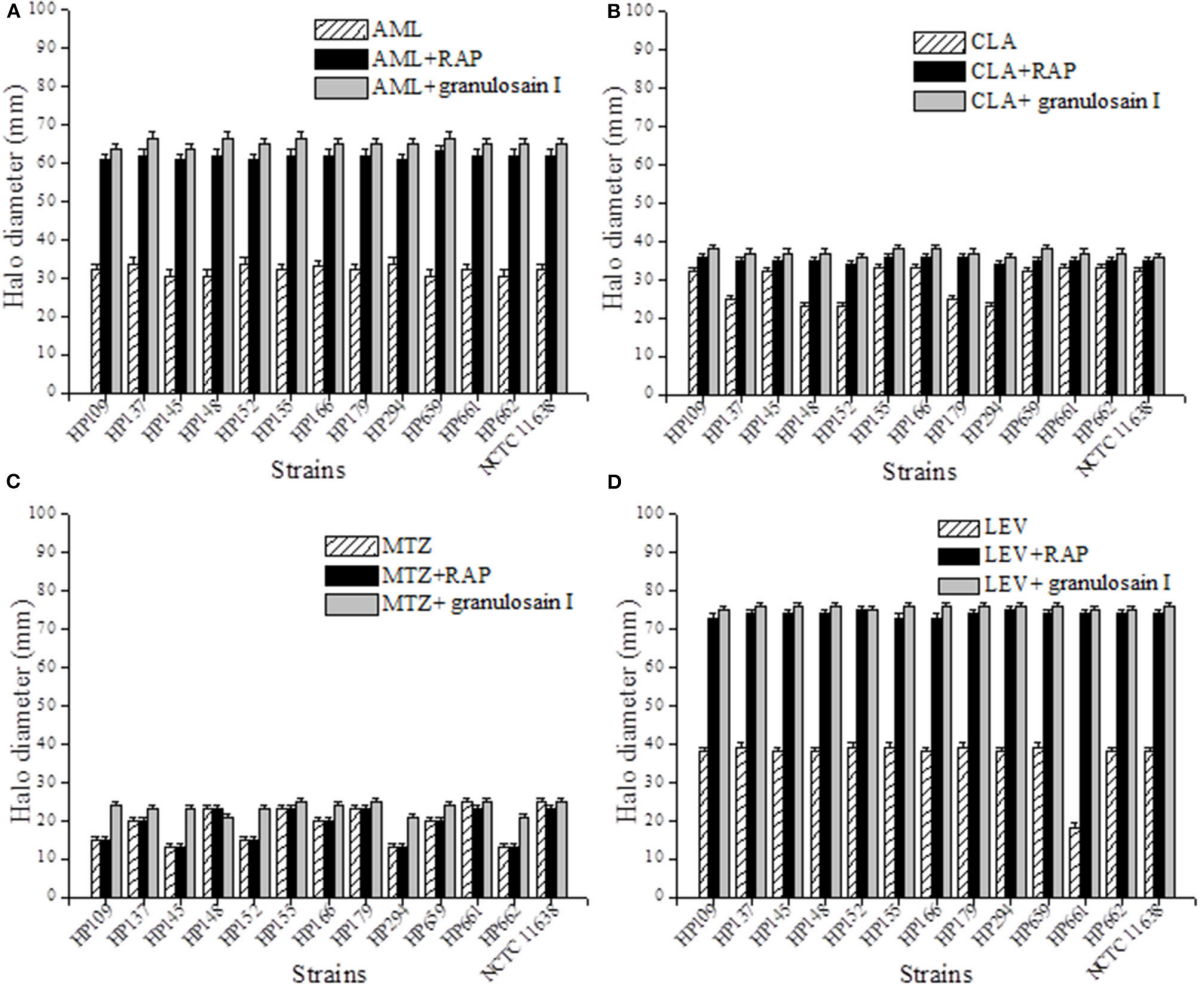
Synergistic effect against *Helicobacter pylori* after combining the partially purified proteolytic extract (RAP) of the fruits of *Solanum granuloso-leprosum* or granulosain I and antibiotics such as **(A)** AML, **(B)** CLA, **(C)** LEV and **(D)** MTZ.

Figures 3A–D shows the halo diameters (mm) measured in the cultures of *H. pylori* strains after 3 days of incubation with antibiotic alone and with the combined treatment of the RAP or granulosain I and amoxicillin (AML, 10 μg), clarithromycin (CLA, 15 μg), metronidazole (MTZ, 5 μg), or levofloxacin (LEV, 5 μg).

As shown in Figures 3A–D, the combined synergistic effects of RAP (312.5 mg/mL) or granulosain I (156.25 mg/mL) and several antibiotics commonly used against *H. pylori* strains were demonstrated. The synergistic effect of such combinations goes far beyond each drug alone.

Furthermore, the synergism between the RAP or granulosain I and each antibiotic was evaluated on all *H. pylori* strains at once, as mean of the halo diameters (mm) ± SD, with respect to controls (antibiotic alone). Significant differences (*p* < 0.05) were found between the RAP or granulosain I and AML, LEV, and CLA, using InfoStat/L Statistical Software for Windows (Universidad Nacional de Córdoba, Córdoba, Argentina).

It is important to note the significant differences found between the RAP or granulosain I and LEV with respect to the control, being 73.915 ± 0.705, 75.692 ± 0.484, and 38.5 ± 0.618 mm, respectively. Similarly, significant differences were observed between the RAP or granulosain I and AML with respect to the control, being 61.723 ± 0.719, 65.231 ± 1.033, and 31.954 ± 1.255 mm, respectively. The differences observed between the RAP or granulosain I and CLA with respect to the control were also significant (*p* < 0.05), being 35.154 ± 0.688, 37.077 ± 0.759, and 29.15.5 ± 4.469 mm, respectively.

Therefore, the partially purified proteolytic extract of the fruits from *S. granuloso-leprosum* and granulosain I showed a synergistic combined effect with the antibiotics LEV, AML, and CLA, against the reference *H. pylori* strain and the wild strains isolated from human biopsies. On the contrary, a synergistic combined effect of MTZ and RAP or granulosain I against the *H. pylori* strains was not observed.

These results are particularly relevant for *H. pylori* resistant to LEV (HP 661), CLA (HP 137, HP 138, and HP 139) and potentially useful against multidrug-resistant strains such as HP 152 and 294. Furthermore, all strains assayed are sensitive to AML but the combination of the enzyme extracts and AML allowed to increase the diameter of the halo, indicating that RAP and granulosain I can act as adjuvants, which would allow the recommended dose of AML to be decreased.

According to recent reports, some flavonoid compounds have shown synergism when combined with anti-*H. pylori* first-line antibiotics (63, 64).

### Cytotoxicity Assays

The toxicological effect of RAP and granulosain I was evaluated in BALBc (WT) mice of 20 g of body weight (40 days old), at the maximal MIC values obtained for *H pylori* strains.

The first group of animals was treated with 1 × PBS and named control group. The second group was treated with three doses of 250 μl of RAP (625 μg/mL) at intervals of 48 h. The third group was treated with three doses of 250 μl of granulosain I (312.5 μg/mL) at intervals of 48 h. All animal groups were maintained with food and water *ad libitum*. After three days, the animals were killed and a serum pool was collected. The activities of transaminases and creatinine, enzymes involved in liver and kidney function, respectively, were determined by the Wiener Lab test (Rosario, Santa Fé, Argentina).

**Figure 4:**
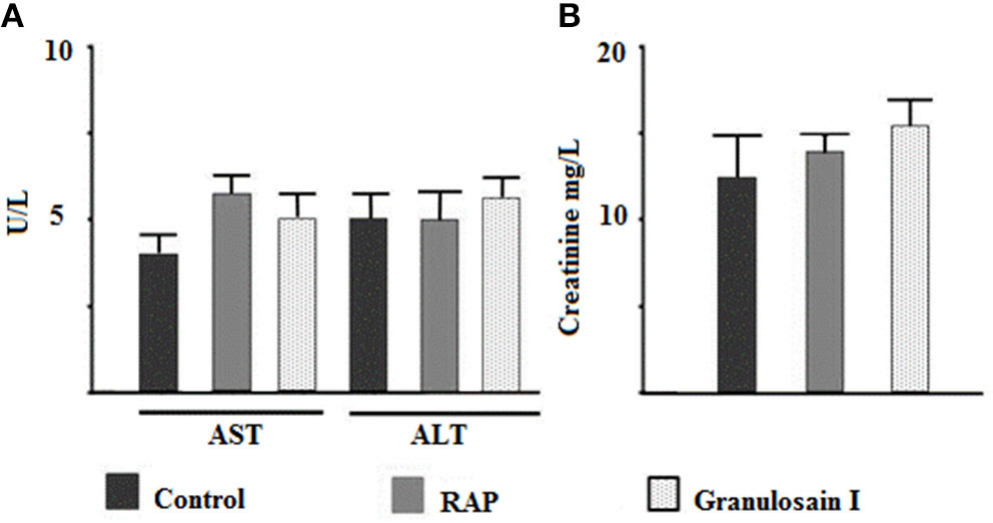
Citotoxicity assays. Effect of the partially purified proteolytic extract (RAP) of the fruits of *Solanum granuloso leprosum* and the main purified fraction (granulosain I) on: **(A)** The activity of aspartate aminotransferase (AST) and alanine aminotransferase (ALT), and **(B)** Creatinine in serum. Results represent the Mean ± SD of three independent experiments. PBS was administered to the control group.

As shown in Figure 4, no significant differences (*p* < 0.05) were observed in the activities of transaminases or creatinine with respect to the control group. Consequently, the partially purified proteolytic extract of the fruits from *S. granuloso-leprosum* and granulosain I did not show toxicological effects at the concentrations studied.

## Conclusions

The dramatic increase in antimicrobial-resistant *H. pylori* strains has alerted the World Health Organization about the need to find new strategies to improve their eradication. Combining prevention and therapeutic treatments seems to be the way to go.

Natural compounds are an interesting alternative because they could increase the effectiveness of conventional therapeutic treatments, and they do not generate microbial resistance. At the same time, the development of functional foods containing safe natural antimicrobial compounds would contribute to the prevention of *H. pylori* infection. Proteolytic enzymes are widespread in nature and play a fundamental role in defending living organisms from bacterial attacks.

This work was focused on the study of the partially purified proteolytic extract (RAP) of the fruits from *Solanum granuloso-leprosum* (Dunal) and the main purified fraction (granulosain I) as safe natural antibacterial agents against *Helicobacter pylori* to be applied as safe food additives for anti-*H. pylori* functional foods and as natural adjuncts to conventional therapies.

In this study, the antibacterial effects of the RAP and granulosain I against *H. pylori* strain, sensitive to AML, LEV, CLA, and MTZ, single-drug resistant to LEV, CLA, and MTZ, and multidrug resistant to CLA and MTZ, have been demonstrated. Granulosain I and the RAP were able to inhibit the microbial growth of most strains (11 of 13) at concentrations as low as 312.5 and 625 μg/mL, respectively, whereas in the other two strains, these values were doubled.

The antipathogenic effect of the RAP and granulosain I against crucial genes encoding pathogenic factors, such as *omp*18, *ure*A, and *fla*A (whose expression is relevant for *H. pylori* to colonize the gastric mucosa), has been revealed. This study contributes to the search of novel natural antibiotics which act on specific *H. pylori* targets that include pH control (urease activity) and adherence pathways (OMPs), and secretion and activity of pathogenic factors.

The synergistic combined effect of the RAP and granulosain I with AML, CLA, and LEV has been also showed. Consequently, the RAP and granulosain I could decrease the antibiotic side effects or prevent the emergence of new antibacterial resistance among *H. pylori* strains.

Finally, the relevant physicochemical and antimicrobial properties of the RAP and granulosain I will allow the formulation of safe functional foods with anti-*H. pylori* activity. However, further studies are needed to elucidate the mechanism of action of these compounds and to evaluate their pharmacokinetics.

## Data Availability Statement

The original contributions presented in the study are included in the article/supplementary material, further inquiries can be directed to the corresponding author/s.

## Author Contributions

AV and SB designed the experiments and did the data analyzing and manuscript writing. ÁS and MA did the experimental assays, data collection, and analysis. DV collaborated with ÁS and MA in the preparation of partially purified enzymatic extracts and purified fraction. All authors contributed to the article and approved the submitted version.

## Funding

This work was supported by the National University of San Luis, San Luis, Argentina (Grant Nos. 2-0718 and 02-4118). ÁS and MA are Postdoctoral Fellows from CONICET, Argentina. SB is Researcher Career Member at CONICET, Argentina.

## Conflict of Interest

The authors declare that the research was conducted in the absence of any commercial or financial relationships that could be construed as a potential conflict of interest.

## Publisher's Note

All claims expressed in this article are solely those of the authors and do not necessarily represent those of their affiliated organizations, or those of the publisher, the editors and the reviewers. Any product that may be evaluated in this article, or claim that may be made by its manufacturer, is not guaranteed or endorsed by the publisher.
