# Supplementary material for: Antimicrobial Effect of a Proteolytic Enzyme From the Fruits of Solanum granuloso-leprosum (Dunal) Against Helicobacter pylori
Source: Front Nutr. 2021 Dec 16;8:699955. doi: 10.3389/fnut.2021.699955 (PMC8717831; doi:10.3389/fnut.2021.699955)
Supplement: Supplementary file 1 [file Table_1.doc]

Table S1. Antibiotic sensitivity of *Helicobacter pylori* strains.

| *Helicobacter pylori* strain | Pathology | Amoxicillin (AML) | Clarithromycin (CLA) | Metronidazole (MTZ) | Levofloxacin (LEV) |
| --- | --- | --- | --- | --- | --- |
| NCTC 11638 |  | Sensitive | Sensitive | Sensitive | Sensitive |
| HP 155 | Chronic gastritis | Sensitive | Sensitive | Sensitive | Sensitive |
| HP 166 | Chronic gastritis | Sensitive | Sensitive | Sensitive | Sensitive |
| HP 659 | Chronic gastritis | Sensitive | Sensitive | Sensitive | Sensitive |
| HP 137 | Chronic gastritis | Sensitive | Resistant | Sensitive | Sensitive |
| HP 148 | Gastric ulcer | Sensitive | Resistant | Sensitive | Sensitive |
| HP 179 | Chronic gastritis | Sensitive | Resistant | Sensitive | Sensitive |
| HP 109 | Chronic gastritis | Sensitive | Sensitive | Resistant | Sensitive |
| HP 145 | Duodenal ulcer | Sensitive | Sensitive | Resistant | Sensitive |
| HP 662 | Chronic gastritis | Sensitive | Sensitive | Resistant | Sensitive |
| HP 661 | Gastric ulcer | Sensitive | Sensitive | Sensitive | Resistant |
| HP 152 | Gastric ulcer | Sensitive | Resistant | Resistant | Sensitive |
| HP 294 | Duodenal ulcer | Sensitive | Resistant | Resistant | Sensitive |
